# Computer Simulations of Soft Responsive Gels with Embedded Regular Arrangements of Stiff Fibers

**DOI:** 10.1021/acs.langmuir.5c04676

**Published:** 2026-01-13

**Authors:** Victor V. Yashin, Santidan Biswas, Anna C. Balazs

**Affiliations:** Chemical Engineering Department, 6614 University of Pittsburgh, Pittsburgh, Pennsylvania 15261, United States

## Abstract

Designing polymeric composites that enhance the material’s resilience and mechanical properties is vital in the production of such technologically important systems as aeronautic components, biomimetic architectures and thin film displays. Taking advantage of the rich deformation behavior exhibited by regular, geometric arrangements of stiff fibers, we used computer simulations to analyze the properties of composites formed by embedding a layer of fibers, arrayed into rectangular, hourglass and honeycomb structures, into the middle of a thicker hydrogel. We determined how the geometry of fiber layer affected the composite’s resistance to finite deformations and changes in shape. Our computer simulations revealed cooperative interactions between the embedded stiff fibers and the gel matrix that led to mechanical reinforcement under both small and finite shear and tensile deformations, and to shape changes under finite tensile deformations. The latter effects depended on a mismatch between the Poisson’s ratio of the gel matrix and fiber geometry of the fiber layer. When the Poisson’s ratio for the fiber layer and the gel were both positive and comparable in value, the composite did not undergo significant shape change, as evidenced in the case involving the rectangular fiber arrangement. The computer simulations showed that for particular fiber arrangements (hourglass and honeycomb), the composite could exhibit the auxetic behavior (negative Poisson’s ratio) in one or two directions. Our results yield design rules for enhancing the resilience and mechanical properties of the polymer matrices and thus yield superior composites for technological applications.

## Graphical abstract

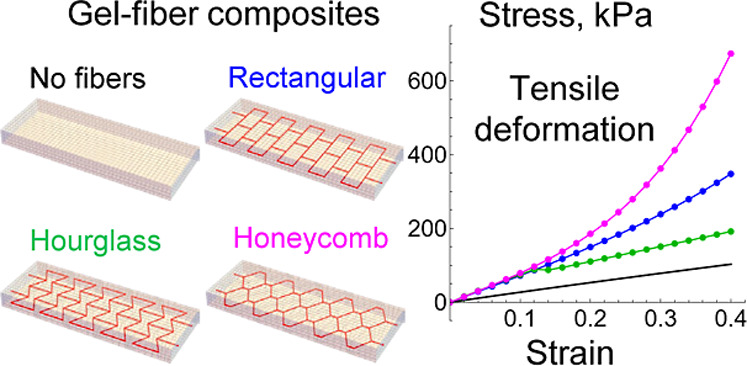

## Introduction

When a rigid cellular solid is combined with a soft matrix, the resulting cellular material generally exhibits material properties that are significantly different from those of the constituent parts.^1–3^ When the two constituent components of the material are considered separately, the basic features of their mechanical behavior are well understood and can be predicted using theoretical models. In the case of hydrogel-fiber composites, however, their properties, and particularly the auxetic behavior of the composite is more challenging to predict a priori. While the behavior of hard–soft cellular materials, composed of thin-walled cellular structures filled with an elastomer or polymer gel have been well-studied,^1–7^ the behavior of cellular composites considered here are less well-known. We use computer simulations to examine the mechanical properties of a composite material composed of a soft thermoresponsive hydrogel and an embedded layer of thin, stiff fibers, which are arranged in the ordered cellular structure shown in Figure 1. We focus on symmetric systems where the fiber layer is embedded in the center of the gel to reduce the effects of out-of-plane deformations. We demonstrate that the type of fiber arrangement in the hydrogel strongly affects both the material’s resilience and reinforcement The simulations also reveal fiber arrangements that enable the composite to exhibit the auxetic behavior (negative Poisson’s ratio) in one or two directions. Consequently, if stretched in the horizontal direction, the material will expand in a transverse direction (as opposed to a shrinking along that direction, as observed for materials with a positive Poisson’s ratio) and thereby effectively increase the structure’s volume. The auxetic cellular structures are known to enhance the material’s resistance to impact and provides greater protection against crashes for automotive applications, greater structural stability of buildings of importance to the construction industry and greater flexibility for stents and implants used for medical applications.^8^ Establishing new design rules for producing auxetic materials is significant to advances in a range of technological applications. Here, we show that embedding layers of regularly arranged stiff fibers into a soft hydrogel matrix produces gel composites with unique mechanical properties and unexpected responses to deformation, including the remarkable auxetic behavior.

**Figure 1:**
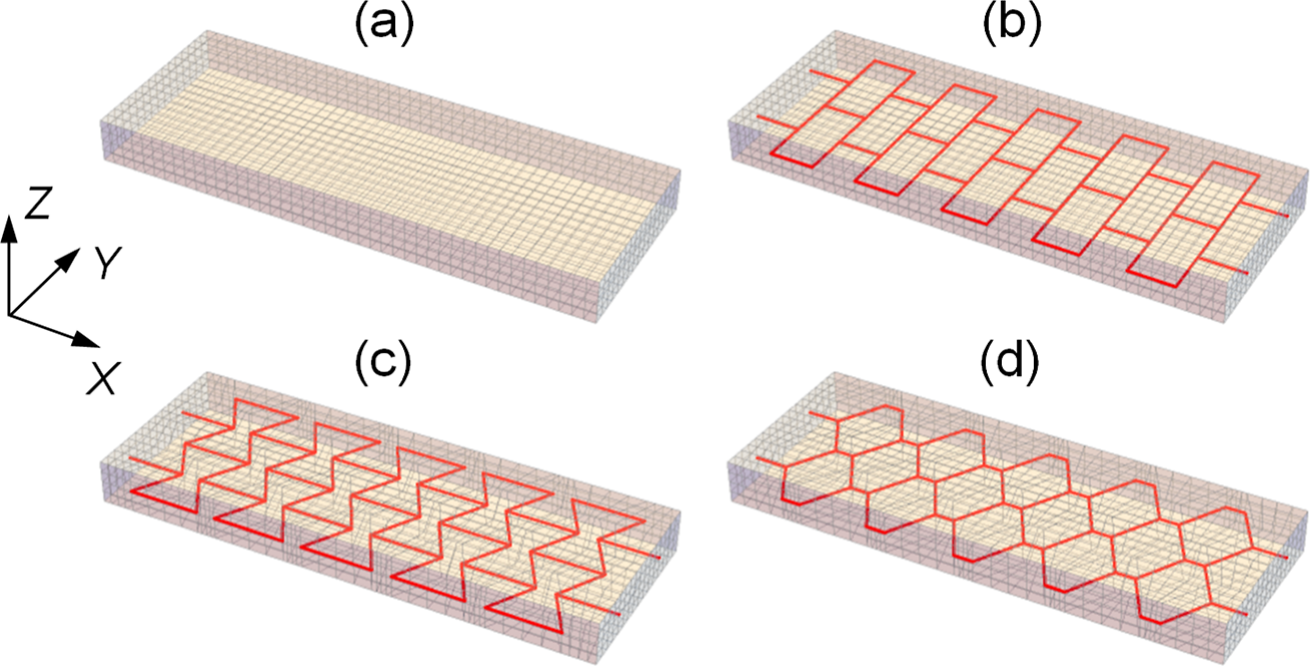
Gel samples used in the computational modeling. (a) Pure gel, and gel samples having a fiber layer of (b) rectangular, (c) hourglass, and (d) honeycomb arrangement embedded in gel’s middle cross-section. Mesh size is 44 × 16 × 4 Δ_0_^3^ in the *XYZ* directions, where Δ_0_ = 1 mm is the computation length scale.

## Materials and Methods

To start, we describe characteristics of the individual constituents and then detail the behavior of the cellular gel-fiber materials in the Results and Discussion section. For the stiff cellular structures like the fiber layers in Figure 1b–d auxetic behavior under tensile deformations can be controllably introduced through varying the geometry of a repeating cell.^9–15^ Figure 2a shows the three square unit cells, which differ in the angle between the four fibers and the *Y* axis. The arrangement of fibers within the square unit cell is rectangular at the angle of 0°, hourglass at −30° and honeycomb-like at +30° (Figure 2a). Assuming the fibers to be inextensible and freely jointed at the points of connection, a purely geometric consideration can be used to calculate a relative change in the cell size along the axis *Y*, i.e., the strain ε_*yy*_, resulting from an extension or contraction of the cell along the axis *X* characterized by the tensile strain ε_*xx*_.^16,17^

**Figure 2:**
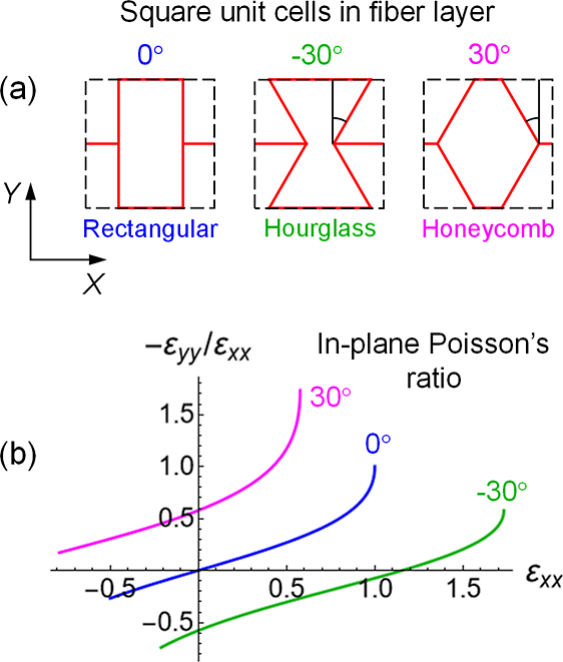
(a) Square unit cells and (b) in-plane Poisson’s ratios −ε_*yy*_/ε_*xx*_ of flat, freely jointed fiber layers having rectangular (blue) hourglass (green) and honeycomb (red) arrangements. The plots show the behavior the fiber arrangements alone, without the presence of the gel.

The quantity (−ε_*yy*_/ε_*xx*_) indicates the value of the Poisson’s ratio for the material, which characterizes the deformation (expansion or contraction) of a material in directions perpendicular to the specific direction of loading. The ratio represents the relative contraction in one direction to relative expansion of a material in another direction. The Poisson’s ratio is positive for typical solids.^18^ In the case of three of the cellular structures considered here, however, the sign of Poisson’s ratio is positive for only one of the arrangements, the honeycomb structure in Figure 2b. In contrast, the hourglass arrangement of fibers is auxetic, i.e., it exhibits negative Poisson’s ratio in a wide range of tensile strains (green line in Figure 2b). Hence, unlike a typical solid material, the cellular material with a hourglass structure extends in *Y* when extended in *X* and contracts in *Y* when contracted in *X*. As for the rectangular arrangement of fiber, the Poisson’s ratio is zero at infinitesimal deformations, positive at ε_*xx*_ > 0 and negative at ε_*xx*_ < 0 (see the blue-colored line in Figure 2b).

The soft hydrogel matrix in the gel-fiber composites modeled here is assumed to be a poly­(*N*-isopropylacrylamide) gel (NIPA gel), which is a well characterized, widely used thermoresponsive polymer network. A NIPA gel exhibits a lower critical temperature (LCST) behavior, i.e., it is swollen in water at temperatures *T* below the critical one of *T*_c_ ≈ 33 °C and undergoes a transition to the collapsed (unswollen) state at *T* > *T*_c_.^19^ The state of swelling and equilibrium mechanical properties of a NIPA gel can be calculated by balancing the elastic stress according to the neo-Hookean model of polymer network elasticity, σ_el_ = *c*_0_λ^–3^(λ^2^ – 1/2), and the osmotic pressure of monomeric units according to the Flory–Huggins theory π_FH_ = – [ϕ + ln­(1 – ϕ) + χ­(ϕ,*T*)­ϕ^2^].^19–21^ Here, *c*_0_ is the dimensionless cross-link density in the as-prepared polymer network, λ is the degree of swelling, ϕ the volume fraction of polymer in gel, and the function χ­(ϕ,*T*) describes the polymer–solvent interactions. Under isotropic swelling, the volume fraction of polymer in the gel, ϕ, is related to the degree of swelling λ as ϕ = ϕ_0_λ^–3^, where ϕ_0_ is the volume fraction of polymer in the undeformed (as-prepared) gel.

Figure 3 shows the characteristics of pure NIPA gel calculated for the model parameters used in this study. Specifically, we assume that *c*_0_ = 1.08 × 10^–3^ and ϕ_0_ = 0.129, and the polymer–solvent interaction function is taken from the literature χ­(ϕ,*T*) = 3.416 – 902.441/*T* + 0.518ϕ.^19^ The unit of pressure is σ_0_ = 135 MPa in the study. The swelling–deswelling transition at approximately 33 °C is clearly seen in Figure 3a, where the equilibrium degree of swelling, λ_eq_, is displayed as a function of the temperature, *T*. Figure 3b shows that the polymer content within the gel under the equilibrium swelling, ϕ_eq_, increases with an increase in the temperature. As the gel becomes denser with an increase in temperature, the shear, *G*_0_, and Young’s, *E*_0_, elastic moduli of gel also increase (see Figure 3c).^22^ According to the neo-Hookean model of elasticity, the dimensionless shear modulus of gel as a function of the temperature *T* is calculated simply as *G*_0_(*T*) = *c*_0_/λ_eq_(*T*). Here, *c*_0_ is the cross-link density of polymer network and λ_eq_(*T*) is the equilibrium degree of swelling shown in Figure 3a.

**Figure 3:**
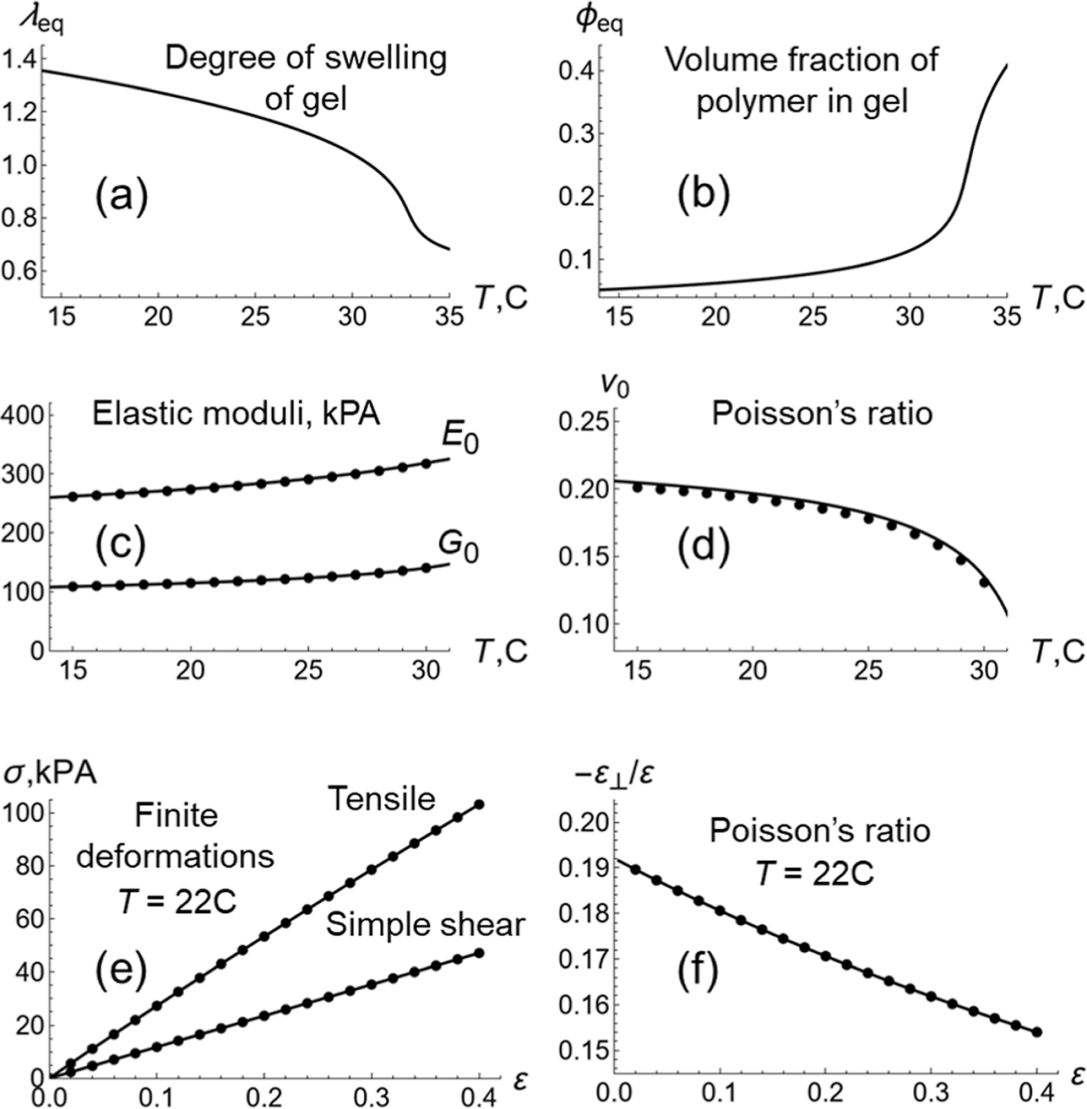
Mechanical properties of pure NIPAAm hydrogel obtained from theory. Equilibrium (a) degree of swelling of gel λ_eq_, (b) volume fraction of polymer in gel ϕ_eq_, (c) shear *G*_0_ and Young’s *E*_0_ moduli of gel, and (d) Poisson’s ratio ν_0_ of gel as functions of temperature *T*. (e) Tensile and simple shear stresses σ and (f) Poisson’s ratio −ε_⊥_/ε as functions of strain ε under finite deformations at *T* = 22 °C. The symbols in (c–f) show the data obtained in the numerical simulations.

Under infinitesimal deformations, the Poisson’s ratio of the pure gel (plotted in Figure 3d as a function of *T*) is calculated through the shear and Young’s elastic moduli as $\nu_{0}=E_{0}{\left(2G_{0}\right)}^{-1}-1$ according to the linear elasticity theory.^18^ Note that ν_0_ decreases as *T* approaches the critical temperature (Figure 3d). (Experiments show that ν_0_ might become negative under some ϕ_0_ and *c*_0_ ^19^).

Finally, the stress–strain curves (Figure 3e) and Poisson’s ratio (Figure 3f) of a hydrogel can be calculated for finite deformations under swelling equilibrium at a given temperature *T*.^22^ (Throughout the paper, all stresses are true stresses, and all strains are engineering strains.) Note that for finite simple shear deformations of a hydrogel, the neo-Hooken model of polymer network elasticity predicts that the shear stress σ is a linear function of the shear strain ε with the slope being equal to the shear modulus *G*_0_(*T*) given above.^22^

For finite tensile deformations, the Poisson’s ratio is calculated according to the definition ν = −ε_⊥_/ε, where ε is the tensile strain and ε_⊥_ is the strain in the direction normal to the deformation. The theory predicts that the Poisson’s ratio decreases monotonically as a function of finite ε tensile strain at a given temperature *T* (Figure 3f).

The above calculations provide a basis of comparison for the properties of the gel-fiber composite, which are described below.

## Results and Discussion

When a layer of fibers is ordered into a cellular structure and embedded in a hydrogel, the mechanical properties of the resulting composite material cannot be analyzed using a combination of simple theoretical tools such as those described above. To this end, we utilize our computational model that was developed to study shape changes of hydrogel membranes with stiff fibers attached to their surface.^23,24^ The hydrogel is simulated using the gel lattice spring-model (gLSM), which is an explicit finite element method. The gel is represented by trilinear hexahedral elements with the nodal points moving due to the elastic and osmotic forces under the assumption that the gel dynamics is purely relaxational.^25–27^ The size and shapes of the gel elements described herein are chosen to accommodate a given arrangement of fibers (see Figure 1b–d). Figure 3c–f show that the gLSM approach provides an excellent agreement between the theory and simulations.

The fibers are assumed to exhibit linear elastic behavior. Each fiber is modeled by a sequence of straight segments placed along the edges of gel elements. The fiber nodes are attached to and move together with the gel nodes. We assume perfect adhesion between the fibers and the hydrogel matrix, and disregard debonding (that could occur in physical samples under finite deformation). The nodal forces exerted by the gel matrix under deformation are calculated from the energy density of the gel, *u* = *u*_el_ + *u*_FH_, where *u*_el_ is the neo-Hookean elastic energy density and *u*_FH_ is the Flory–Huggins energy density of the polymer–solvent interactions.^25–27^

Due the presence of fibers, the nodal elastic forces include two contributions from the fiber layer. The first contribution is due to the stretching and bending deformations of the fibers. The second contribution originates from the deformation of the hinges, the segments that interconnect different fibers. Figure 4a shows the rectangular arrangement of fibers where the fiber segments forming a hinge are marked in blue.

**Figure 4:**
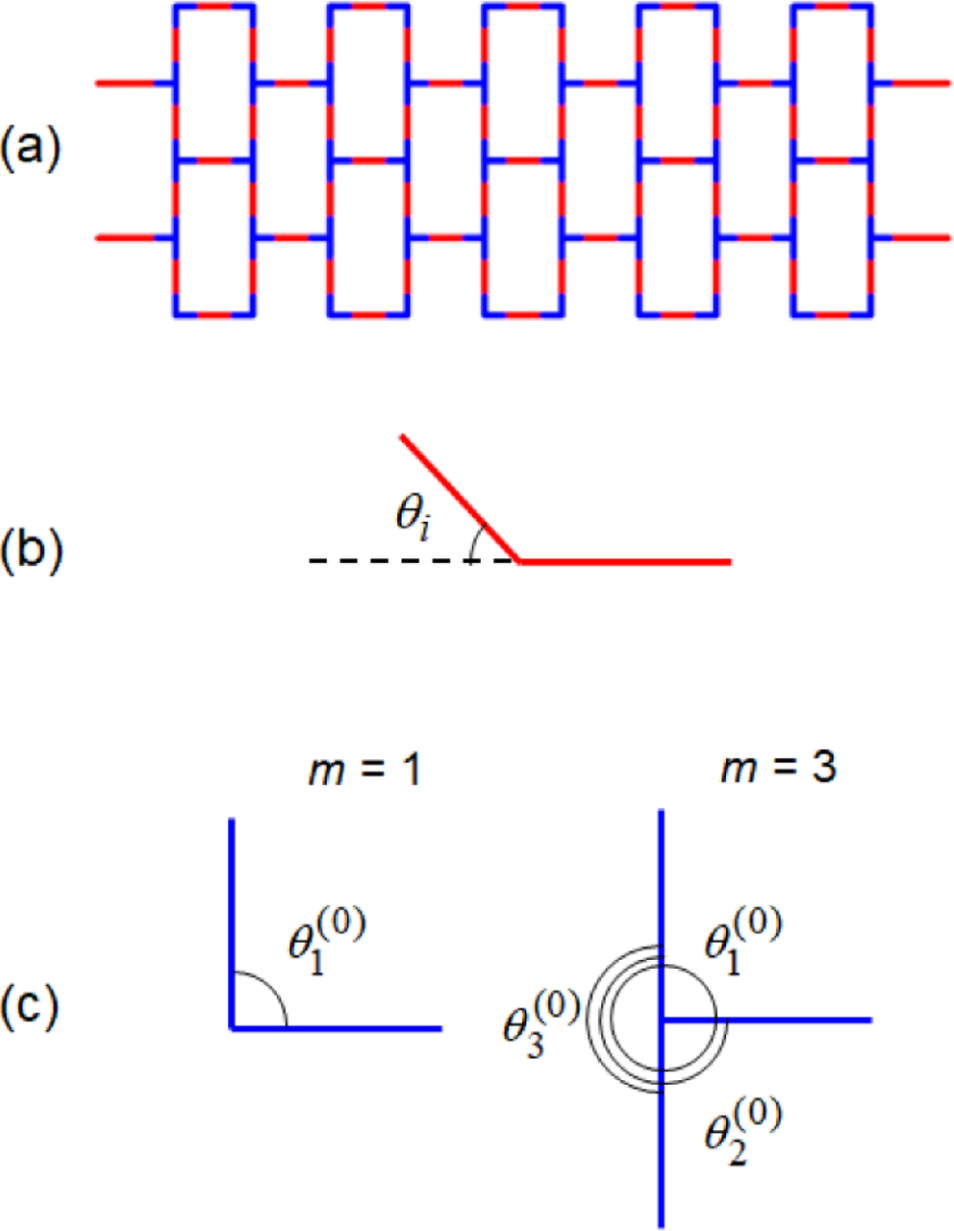
(a) Fiber layer with hinges marked in blue, and schematics of angles in calculations of the elastic energies of (b) fiber and (c) hinge.

The elastic energy of a fiber is calculated as

1 $$U_{f}=\frac{k_{s}}{2}\sum_{n}{\left(l_{n}/l_{0}-1\right)}^{2}+2k_{b}\sum_{i}\frac{1-\cos\theta_{i}}{1+\cos\theta_{i}}$$

The first term describes the energy of stretching the fiber segments, which are characterized by the stretching stiffness *k*_s_, where *l*_*n*_ is the length of the segment *n* of the fiber and *l*_0_ is the equilibrium segment length (note that *l*_0_ can differ from fiber to fiber). The second term in eq 1 is the fiber bending energy, which is defined by the bending stiffness *k*_b_ and depends on the angles between two consecutive segments θ_*i*_, as illustrated in Figure 4b. We use the equation involving a restricted bending (ReB) potential^28^ for the efficient numerical implementation of the model. The ReB potential reproduces the linear elasticity theory in the limit of θ_*i*_ → 0,^18^ when the bending energy is quadratic with respect to the bending angles.

The stretching and bending elasticity constants in eq 1 are related to the Young’s modulus of the fiber material *E*_f_ as *k*_s_ ∼ *E*_f_ *a* ^2^ *l*_0_ and *k*b ∼ *E*_f_ *a* ^4^ *l*_0_^–1^, respectively,^18^ where *l*_0_ is the length of a fiber segment in eq 1 and *a* is the fiber thickness. In the simulations, each fiber in the system consists of four equal segments. In the case of rectangular arrangement of fibers (Figure 2a), all fibers in the system have the same length 4Δ_0_ and hence, the elasticity parameters *k*_s_ and *k*_b_ are the same for all fibers. We take *k*_b_ = *c*_0_ and *k*_s_ = 10*k*_b_ in the latter case, where *c*_0_ is the cross-link density of gel. For the parameters used here, the Young’s modulus corresponds to *E*_f_ ∼ 1.5 MPa and the fiber cross-section is *a* ∼ 0.316 mm for a fiber of length 4Δ_0_ = 4 mm. For the hourglass and honeycomb fiber arrangements (Figure 2a), the elasticity parameters *k*_s_ and *k*_b_ are assigned to each individual fiber according to the relative fiber length *l*_0_/Δ_0_.

If the fibers are freely jointed, the hinges do not exert additional elastic forces. Here, we consider elastic hinges, i.e., fiber–fiber junctions with controllable elasticity. Specifically, we assume that variation of angles between the fiber segments, which are connected in a hinge, has an energy cost given by the ReB potential^28^

2 $$U_{h}=\frac{k_{h}}{2}\sum_{i=1}^{m}\frac{{\left(\cos\theta_{i}-\cos\theta_{i}^{\left(0\right)}\right)}^{2}}{\sin^{2}\theta_{i}}$$

where *k*_h_ is the hinge elasticity parameter. In the above equation, *m* = 2 or 3 corresponds to the number of arms in a hinge, and θ_*i*_ and θ_*i*_^(0)^ are the respective actual and equilibrium hinge angles (see Figure 4c). The functional form of hinge energy function in eq 2 is used only if θ_*i*_^(0)^ ≠ π. In the limit θ_*i*_^(0)^ → π, the hinge energy function in eq 2 is calculated as $2k_{h}\left(1+\cos\theta_{i}\right){\left(1-\cos\theta_{i}\right)}^{-1}$ . As in the case of bending, the ReB potential in eq 2 is quadratic with respect to δθ_*i*_ = θ_*i*_ – θ_*i*_^(0)^ at small deviations from equilibrium. The hinge elasticity *k*_h_ is an adjustable parameter and we take *k*_h_ = *k*_b_ in the simulations.

### Mechanical Properties and Poisson’s Ratios at Small Deformation

To obtain the Poisson’s ratios characterizing the composite, we must first calculate the strain in the system due to tensile and shear deformation. The latter calculations also allow us to determine the fiber structures that provide the greatest resistance to the deformation and thus provide optimal reinforcement. Analogously, we can determine the fiber structures that are the most resistant to shape change. Notably, when the fillers are uniformly distributed in a polymer matrix, an increase in reinforcement is accompanied by an increase in shape stability. In our case, however, the fillers (fibers) are not uniformly distributed within the gel, leading to the distinct structure-dependent behavior observed below.

### Response to Shear and Tensile Deformation

We first examine the mechanical properties of the gel-fiber composites under small deformation as a function of the temperature, *T* (Figure 5). Specifically, we model the simple shear deformations in the *XY* plane and the tensile deformations along the *X* axis at the strains of 0.01 (see Figure S1 in the Supporting Information for schematics). Initially, we fix all surface nodes including the top and bottom surfaces (see Figure S1), so that only internal nodes can move during relaxation. During the tensile deformation, only the *X* coordinates of the nodes on the left and right faces are fixed (see Figure S1), and all other degrees of freedom can relax freely. In the specified temperature range (15–30 °C), the NIPA gel remains in the swollen state.

**Figure 5:**
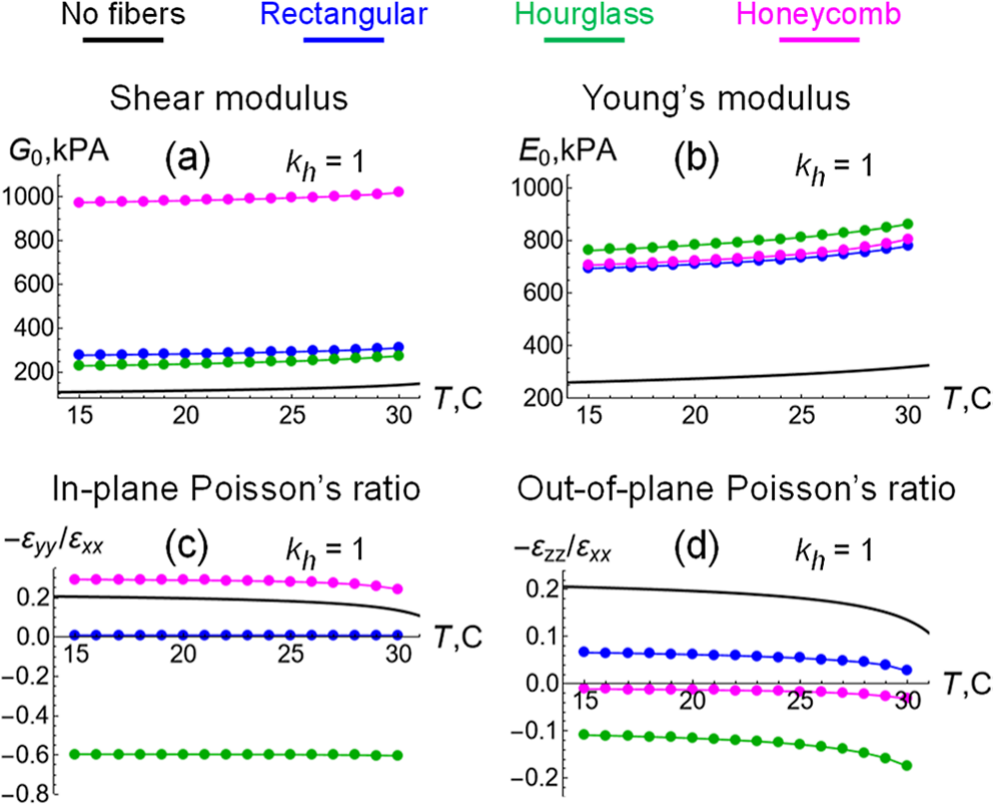
Mechanical properties of gels with fiber layers embedded in the middle cross-section as functions of temperature *T* under small strains of 0.01. (a) Shear modulus *G*_0_, (b) Young’s modulus *E*_0_, (c) in-plane Poisson’s ratio −ε_*yy*_/ε_*xx*_, (d) out-of-plane Poisson’s ratio −ε_*zz*_/ε_*xx*_, are shown for the fiber layers of rectangular (blue), hourglass (green) and honeycomb (magenta) arrangements in the case of elastic hinges at *k*_h_ = *k*_b_. Symbols: data points obtained at small strains of 0.01, connected by lines for better eye guidance. Black lines: calculations for pure gel in Figure 3 for comparison. Note that the Poisson’s ratios for the unit cells in Figure 2b are close to zero, negative ∼−0.5 and positive ∼0.5 for the rectangular, hourglass and honeycomb arrangements, respectively.

The calculated values for both the shear modulus, *G*_0_, and the Young’s modulus, *E*_0_, in Figure 5 are color coded to indicate the specific fiber arrangement. The reference data for the pure gel are shown in black. Higher values of *G*_0_ indicate that the material is more resistant to shear deformation and higher values of *E*_0_ indicate that the material is more resistant to tensile deformation. As seen in Figures 5a,b, the values of *G*_0_ and *E*_0_ for the fiber containing sample are greater than the corresponding values for the gel alone. Hence, the fiber layers provide a degree of reinforcement; the magnitude of the reinforcement, however, depends on fibers’ specific arrangement in the gel.

The honeycomb arrangement of fibers produces the greatest reinforcement as the samples are subjected to simple shear. Indeed, the shear modulus *G*_0_ of the latter composite exhibits an approximately 10-fold increase relative to that in the pure gel (as seen by comparing the magenta and black lines in Figure 5a). While the effects of embedding the rectangular (blue line) and hourglass (green line) shaped layers on *G*_0_ are also significant (∼3.5 times increase), they have a less dramatic effect than displayed by the honeycomb pattern (Figure 5a).

While one arrangement stands out in providing resistance to shear, the reinforcing effects of all three composites are comparable under small tensile deformations, Nonetheless, the Young’s modulus for these three cases are approximately ∼3.5 times greater than the value of *E*_0_ for the pure gel (Figure 5b).

### Poisson’s Ratios for Composite

The three composites, however, do display quantitative differences in their values for the Poisson’s ratio, which indicates how extension of a gel sample along the *X* axis affects the sample’s size in the directions normal to deformation. Since an initially flat fiber layer is embedded in the *XY* plane of a hydrogel slab (see Figure 1), the uniaxial deformation along *X* results in different in-plane (along *Y*) and out-of-plane (along *Z*) deformations of the sample. The corresponding in-plane, −ε_*yy*_/ε_*xx*_, and out-of-plane, −ε_*zz*_/ε_*xx*_, Poisson’s ratios are plotted as functions of the temperature *T* in Figure 5c,d, respectively. As a point of reference, the black lines indicate the Poisson’s ratio of a pure gel (Figure 3d).

Notably, a comparison between Figures 5c and 2b indicates that at small deformations corresponding to ε_*xx*_ → 0, the in-plane Poisson’s ratios of composites with gel of the given thickness (Figure 5c) exhibit qualitatively similar dependence on the fiber arrangement as those for a unit cell (Figure 2b). In both cases, the gel reinforced with fibers in the honeycomb arrangement exhibits a positive in-plane Poisson’s ratio (magenta line), and this ratio is greater than that for the pure gel (black line in Figure 5c). Similarly, the fibers arranged in a rectangular pattern led to an almost zero value of the ratio (blue line) as ε_*xx*_ → 0. Finally, the gel encompassing the hourglass structure of fibers is auxetic for the in-plane direction as −ε_*yy*_/ε_*xx*_ < 0 (green).

For out-of-plane direction, however, the observed behaviors are quite different (Figure 5d). In addition to the gel with embedded hourglass-arranged fibers (green), the gel encompassing the honeycomb-structured fiber layer also exhibits slightly auxetic out-of-plane behavior as −ε_*zz*_/ε_*xx*_ < 0 (magenta) although the latter value is rather close to zero (Figure 5d). In the case of the rectangular arrangement, the out-of-plane Poisson’s ratio is positive, −ε_*zz*_/ε_*xx*_ > 0 at all temperatures (see blue lines in Figures 5c,d).

Under small deformations, the effects observed in Figure 5 of the embedded fibers on the mechanical properties of the gel originate from the concerted action of the two distinctively different structural components, i.e., the hydrogel and fiber layer. The effects of interaction between the constituent components on the mechanical properties of the composite material are further revealed under application of finite deformations, as discussed below.

### Mechanical Properties and Poisson’s Ratios at Larger Deformation

#### Reinforcement of Composite

When we consider gel-fiber composites deformed up to strains of 0.4 at the constant temperature of *T* = 22 °C, we observed rather unexpected results. We remind the reader that the fiber scaffold is embedded in the center of the gel. (The behavior of the system when the fibers are on top of the gel are discussed further below and the Supporting Information). The stress–strain curves for finite shear and tensile deformations are plotted in Figure 6a,b, respectively. Under large strains for both shear and tensile deformations (magenta lines in Figures 6a,b, respectively), the honeycomb arrangement of fibers provides the strongest reinforcement, i.e., the greatest increase in stress relative to that in the pure gel (black lines in Figure 6a,b). This observation is quite surprising since an increase in the rigidity of a fiber network (without the gel) is typically associated with hourglass-like structures.^9–12^ In contrast, here the hourglass fiber arrangement (green lines) provides the smallest increase in the shear and tensile stresses. The reinforcing effect of the rectangular fiber layer (blue lines) lies in between the other two for both types of finite deformations.

**Figure 6:**
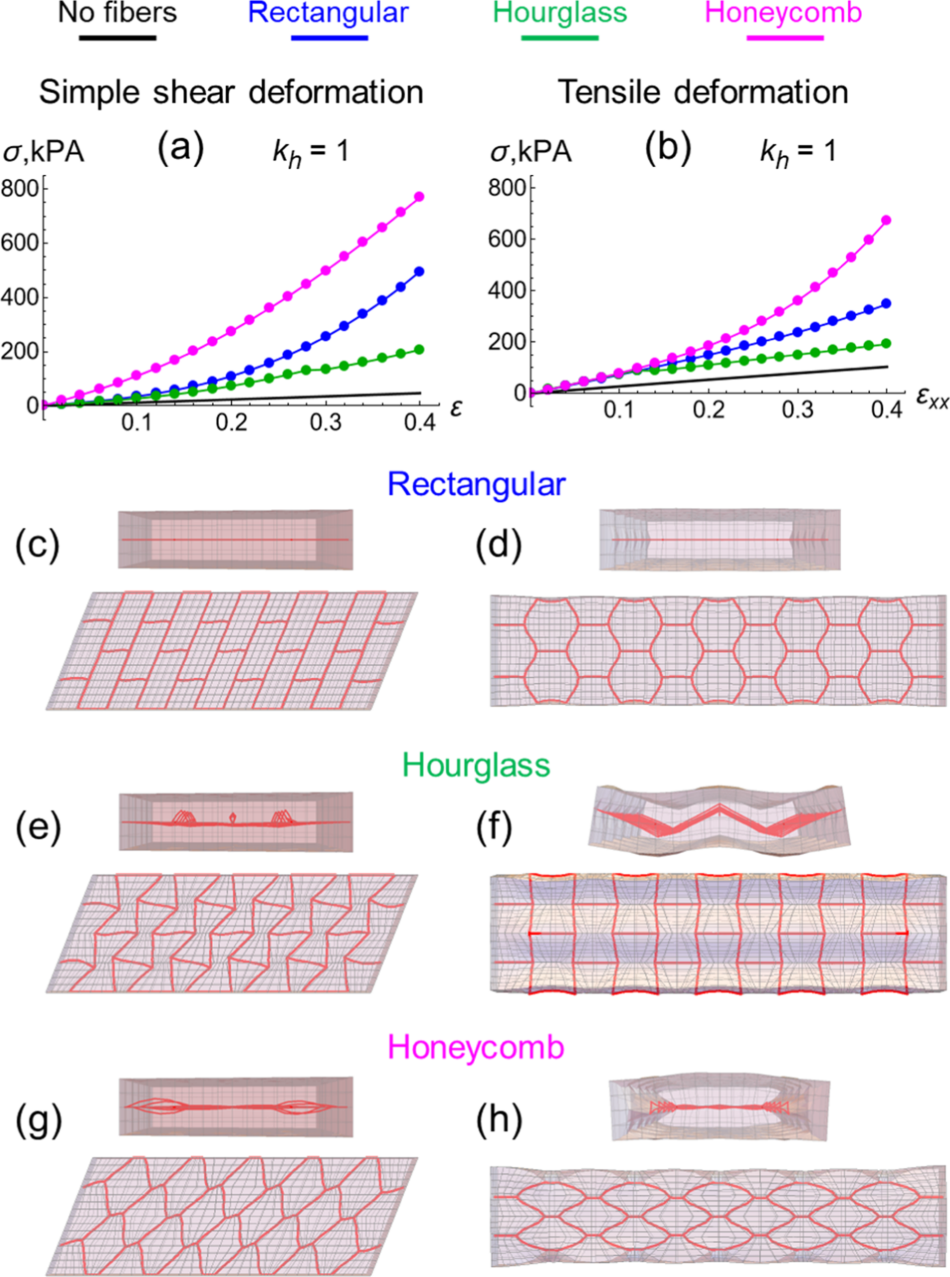
Finite deformations of gels with fiber layers embedded in the middle under the elasticity of hinges *k*_h_ = *k*_b_ at the temperature of *T* = 22 °C obtained by the computer simulations. Stress–strain curves for (a) simple shear and (b) tensile deformations for the fiber layers of rectangular (blue), hourglass (green) and honeycomb (magenta) arrangements, and for pure gel with no fibers (black). Simple shear deformation at the shear strain ε = 0.4: the top and side views of gels for (c) rectangular, (e) hourglass and (g) honeycomb arrangements of fibers. Tensile deformation at the tensile strain ε_*xx*_ = 0.4: the top and side views of gels for (d) rectangular, (f) hourglass and (h) honeycomb arrangements of fibers. Note the out-of-plane deformation of fibers seen in the side views of the gel samples in (e–h).

#### Shape Changes of Composite

Figure 6c–f show the gel samples under finite shear and tensile deformations for the three types of fiber arrangements. Only the rectangular arrangement (Figure 6c,d) does not change shape with deformation. We anticipate that fiber arrangement providing the least reinforcement (hourglass in Figure 6) would be the most vulnerable to changing shape in response to an applied force. This property is indeed seen for the behavior of the composite with hourglass scaffold, which shows a pronounced buckling (Figures 6e,f). The latter shape change can be attributed to the significant mismatch between the Poisson’s ratios of the pure gel and the fiber layer. When the pure gel sample in Figure 1a undergoes extension, the size of gel in the direction normal to that deformation decreases, yielding a positive Poisson’s ratio ∼0.2, as seen in Figure 3f. In contrast, for the hourglass scaffold alone, with the same lateral dimensions as the gel, the size of the scaffold increases under extension and yields a negative Poisson’s ratio of ∼−0.5, (see green line in Figure 2b). Consequently, an hourglass scaffold embedded in a gel with a positive Poisson’s ratio, experiences compressive stress from the gel matrix due to the compression of the matrix in the *Y* direction as the sample extends in the *X* direction. When the compression exceeds some threshold, the fiber layer buckles. This out-of-plane deformation occurs to reduce the elastic energy. In turn, the out-of-plane deformation of the fiber causes deformation of the surrounding soft hydrogel matrix, as seen in Figure 6f. For certain values of the strain, the hourglass and honeycomb fiber arrangements exhibit out-of-plane deformations under both shear (Figure 6e,g) and extension (Figure 6f,h).

Under extension, the composite encompassing the honeycomb scaffold (Figure 6h) also shows a degree of change in shape. (Namely, the sample becomes thicker in the *Z* direction and the shape of composite becomes slightly round at the top in Figure 6h). The latter behavior can also be interpreted in terms of a disparity in the Poisson’s ratios of the soft gel matrix and stiff fiber layer. In this case, the Poisson’s ratio of just the honeycomb scaffold (∼0.5 and greater as shown by the magenta line in Figure 2b) exceeds that of the gel matrix (less than ∼0.2 as seen in Figure 3f). Under extension in the *X* direction, the size of the thin fiber layer in the *Y* direction is expected to decrease faster than that of bulkier the matrix. Therefore, the soft gel matrix experiences an internal compression caused by the embedded stiff fiber layer. As a result, the sample becomes thinner in *Y* and thicker in *Z*, thus acquiring the shape observed in Figure 6h.

The same arguments allow us to explain why the composite encompassing the rectangular scaffold provides the most stable shape. In contrast to the honeycomb and hourglass with fiber layers in the middle in the sample, the centrally located fibers in rectangular composite remains flat up to the maximal strains of 0.4 (see Figure 6c,d). The difference in the Poisson’s ratios of the rectangular fiber layer (blue line in Figure 2b) and gel matrix (Figure 3f) is the smallest among the three composites considered here and decreases with extension. Thus, in this scenario, the most stable shape is achieved when: (1) the fiber layer is in the middle, yielding a symmetric structure, and (2) the matrix and the fiber layer exhibit similar Poisson’s ratios, which indicates similar shape changes of the two constituents under tension.

When the fibers are localized on the top surface of gel slab (Figure S3 in the Supporting Information), the stress–strain behavior remains mainly the same as that displayed in Figure 6a,b. (For fibers attached to the top surface, the nodal coordinates are free to move in all three dimensions on the top and bottom faces of the sample during relaxation). In terms of the shape changes, the sample with fibers on the top layer leads to behavior that is qualitatively like that depicted in Figure 6c–h. The top-coated composites, however, show greater curvature than in Figure 6, as can be seen in Figure S4 in the Supporting Information, reflecting the placement of stiff components on the outer surface of the more compliant gel.^23^ Note that the top-coated rectangular composite remains nearly flat under extension (see Figure S4a in the Supporting Information).

#### In-Plane and Out-of-Plane Poisson’s Ratios

As shown in Figure 6d,f, the shapes of gel samples that are reinforced with the hourglass and honeycomb scaffolds are no longer brick-like at higher tensile strains. It is nevertheless reasonable to characterize changes in the direction normal to the extension through the effective in-plane and out-of-plane Poisson’s ratios. For the in-plane deformations, we determine the effective in-plane strain as ε_*yy*_ = (*y*_max_ – *y*_min_)/*L*_*y*_ – 1, where *y*_min_ and *y*_max_ are the respective minimal and maximal nodal coordinates in the *Y* direction, and *L*_*y*_ is the width of undeformed brick-like sample of a gel composite. The effective out-of-plane strain in the *Z* direction is determined similarly as ε_*zz*_ = (*z*_max_ – *z*_*min*_)/*L*_*z*_ – 1. The effective in-plane and out-of-plane Poisson’s ratios are calculated as usual, i.e., as −ε_*yy*_/ε_*xx*_ and −ε_*zz*_/ε_*xx*_, and are plotted in Figure 7a,b, respectively. As in the figures above, the results for different types of fiber arrangements are color coded; as a reference, the black line represents the Poisson’s ratio of the pure gel (Figure 3f).

**Figure 7:**
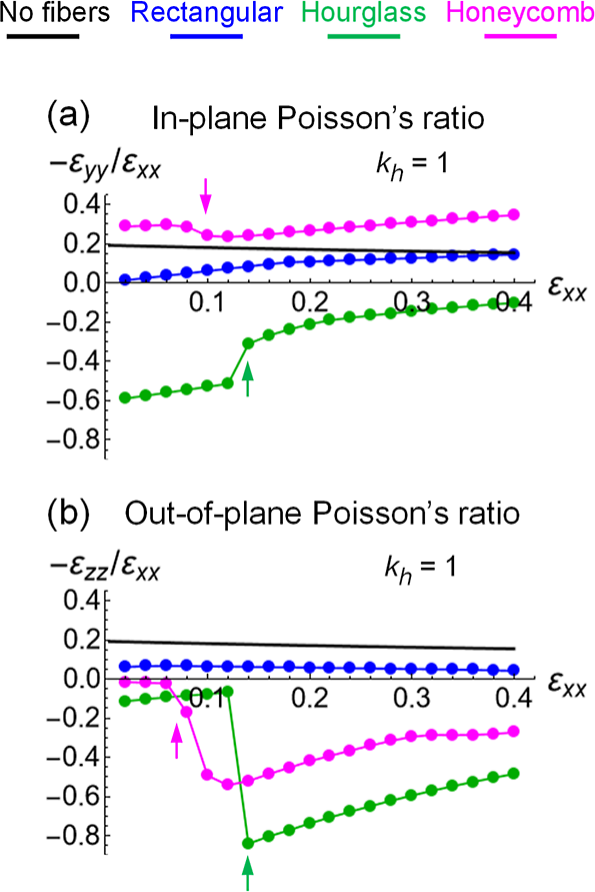
(a) In-plane −ε_*yy*_/ε_*xx*_, and (b) out-of-plane −ε_*zz*_/ε_*xx*_, Poisson’s ratios of gels containing fiber layers embedded in the middle as functions of tensile strain ε_*xx*_ at the hinge elasticity *k*_h_ = *k*_b_ and temperature of *T* = 22 °C. Poisson’s ratios are shown for the fiber layers of rectangular (blue), hourglass (green) and honeycomb (magenta) arrangements. Black: theoretical calculations for pure gel with no fibers in Figure 3f. Arrows mark the onset of out-of-plane deformations of gels having the hourglass (green) and honeycomb (magenta) arranged fiber layers seen in Figure 6e,f.

Figure 7 shows that only the gel composite reinforced with the rectangular scaffold behaves like a customary solid material. Namely, both the in-plane and out-of-plane Poisson’s ratios for the latter gel composite are positive and change smoothly with an increase in the tensile strain ε_*xx*_. Note also that the two Poisson’s ratios are not equal as the composite material is not isotropic due to the embedded fiber layer (blue lines in Figure 7a,b). In contrast, embedding the other two types of fiber layers into the gel creates a composite material that is auxetic (negative Poisson’s ratio) in both or only one direction.

The gel composite encompassing the hourglass-arranged fibers has both the Poisson’s ratios negative (green lines is Figures 7a,b). On the other hand, the honeycomb scaffold leads to auxetic behavior only in the out-of-plane direction, as evident from the magenta-colored lines in Figure 7a,b. (Note that the auxetic out-of-plane behavior of composites made of a rigid honeycomb structure embedded into a soft matrix was reported before, although for a quite different system^4^).

In addition to the auxetic behaviors, the gel composites encompassing the hourglass and honeycomb fiber layers exhibit a shape change at larger strains due to the out-of-plane deformations of the initially flat fiber layers. The shape changes are signified by the abrupt changes in the Poisson’s ratios with an increase in the tensile strain, as seen from the green and magenta lines and arrows in Figure 7a,b.

It is worth noting that the Poisson’s ratios are merely convenient quantitative measures of a sample shape change during extension. Hence, the basic features of the Poisson’s ratios exhibited in Figure 7 by the three types of gel-fiber composites have the same origin as the shape changes discussed in the previous section: the interaction between two constituents that have different inherent behaviors under extension.

As we show below, the contributions from hinges between the fibers play a significant role in the reinforcement and shape changes of the samples.

#### Contribution from Scaffold Elastic Forces to Mechanical Properties

As noted above for the case of larger deformations, the hourglass structure is auxetic along two dimensions; the honeycomb is auxetic in one direction; and the rectangular structure does not display auxetic behavior. We can gain insight into the relationship between the fiber arrangement and the latter mechanical properties by examining the relative contributions of the gel matrix, fiber layer and the hinges to the elastic forces for these three different cases. More generally, the elastic contributions from the fibers and hinges can reveal how the fiber scaffold acts to reinforce the gel, and allows us to pinpoint which of these components is most affected by the mechanical deformation. To this end, we calculate the *X*-components (see Figure S1 for the axes) of nodal forces due to the gel matrix *F*_*n*_^(*g*)^, fibers, *F*_*n*_^(*f*)^ and hinges, *F*_*n*_^(*h*)^, for the nodes located on the corresponding faces of the sample, as illustrated by the schematics in Figure 8a,b. (Due to the specific fiber layer geometry chosen in Figure 1b,c, there is no contribution from hinges to the tensile nodal forces, i.e. *F*_*n*_^(*h*)^ = 0 in Figure 8b). After summation of the nodal contributions, we obtain the total force acting on a chosen face *F*_tot_ = ∑_*n*_(*F*_*n*_^(*g*)^ + *F*_*n*_^(*f*)^ + *F*_*n*_^(*h*)^), and the force generated solely by the fibers and hinges, *F*_L_ = ∑_*n*_(*F*_*n*_^(*f*)^ + *F*_*n*_^(*h*)^). Figure 8c,d display the force ratios *F*_L_/*F*_tot_, versus strain for the respective simple shear and tensile deformations.

**Figure 8:**
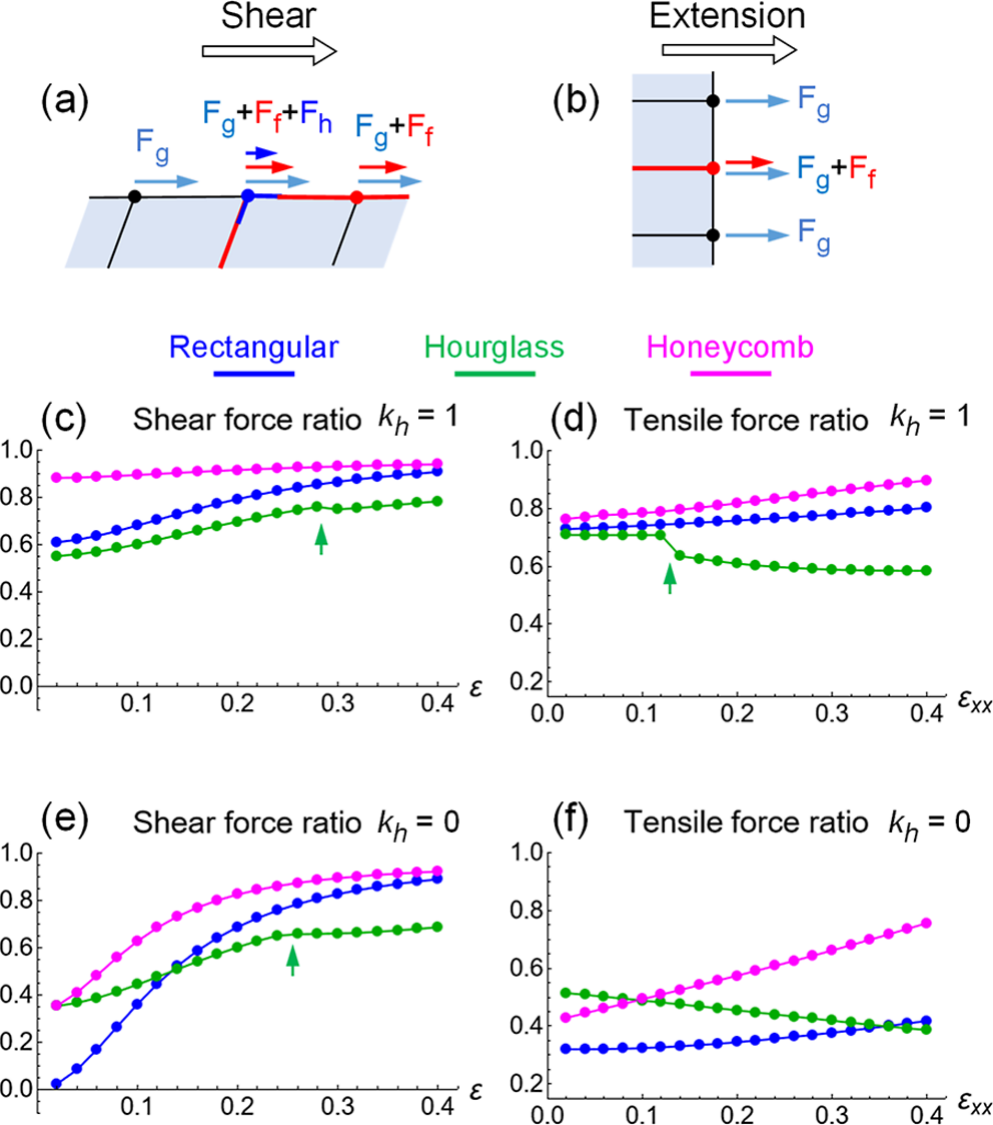
Relative contributions of fibers to the total force under the finite deformations of gel with fiber layers embedded in the middle at the hinge elasticity *k*_h_ = *k*_b_ and *T* = 22 °C. (a) Schematics of calculations and (c) the calculated force ratios for finite simple shear deformations of gels with the fiber layers of rectangular (blue), hourglass (green), and honeycomb (magenta) arrangements. Panels (b,d) show the same as (a,c), respectively, for finite tensile deformations. For comparison, panels (e,f) display the force ratios for finite shear and tensile deformations, respectively, in the case of freely jointed fibers, when the hinge elasticity constant is *k*_h_ = 0. The green colored arrows in (c,e) mark the onset of out-of-plane deformations of hourglass fiber layers in gels under simple shear deformations, and in (d) the green arrow marks the onset of out-of-plane deformations of both fiber layer and gel under extension.

The behavior of the hourglass scaffold (which is auxetic in two directions) is indicated by the green lines in Figure 8c,d; the green arrows mark the onset of the deformations that are observed in Figure 6c,d, respectively. Under simple shear, the transition to the out-of-plane configuration deflects the monotonic increase in *F*_L_/*F*_tot_ (green arrow in Figure 8c), and indicates a decrease in the contribution to the force exerted on the sample from the fibers and hinges (*F*_L_). During extension, the contributions to *F*_L_/*F*_tot_ from the hourglass scaffold drop noticeably after the buckling of the fiber layer (green line in Figure 8d). The decreases in the values of *F*_L_/*F*_tot_ in Figure 8c,d can be understood by recalling that the hourglass scaffold is under compressive stress from the hydrogel matrix due to the mismatch in the in-plane Poisson’s ratios (as discussed above). The transition to the out-of-plane configuration reduces the cost in energy associated with the compression and bending of fibers. (Note that we neglected the effects of the torsional deformations of fibers in the simulations; accounting for fiber torsion might reduce the out-of-plane deformations).

The plots in Figure 8c,d for the elastic hinges (with *k*_h_ = *k*_b_) are consistent with the trends in Figure 6a,b with respect to effect of fiber arrangement on reinforcement (i.e., an increase in stress or force). Namely, the reinforcing effect of the fiber arrangement decreases from the honeycomb to the rectangular to hourglass structures. The figures also reveal that the honeycomb scaffold contributes up to 90% of the elastic force acting on a face at sufficiently high strains (magenta lines in Figure 8c,d). The other two types of scaffolds contribute considerably less, especially to the shear force at lower shear strains (green and blue lines in Figure 8c). Their contributions, however, are greater than 50% for both simple shear and tensile deformations.

The reinforcement due to the fiber arrangements in Figure 8c,d can be attributed to the difference in the length of fibers for these three different arrangements. The honeycomb arrangement encompasses shorter, and the hourglass layout involves longer fibers (Figure 2a) than in those in the rectangular array. Since flexibility of a fiber decreases with the fiber length, the honeycomb scaffold contains the highest number of shorter fibers and is thus the most resistant to deformations (i.e., exhibits greater stresses under given strains). The hourglass cellular structure is the least resistant to deformations. (There is also an effect due the differences in the angles between fibers in a hinge in the three types of arrangements, as seen Figure 2a. The latter effect is likely to be minor because the hinge energy, eq 2, is quadratic with respect to the hinge angle variations under small deformations.)

To obtain further insight into the contributions from the hinge elasticity, we plot the force ratios obtained in the simulations of the simple shear (Figure 8e) and tensile deformations (Figure 8f) in the limiting case of *k*_h_ = 0, i.e., when the fibers are freely jointed at the points of connection. A comparison of Figure 8c,d with Figure 8e,f shows that the contribution from the elastic hinges to the force ratios *F*_L_/*F*_tot_, depends on the type of deformation (shear or tensile) and on the amount of deformation (small or finite). It is worth noting that the freely jointed fibers by themselves (before embedding) exert no force at sufficiently small extension or contraction. Hence, the reinforcing effect of a fiber scaffold at *k*_h_ = 0 (Figure 8f) is solely due to the interaction between the fiber layer and the hydrogel matrix.

For all three scaffold architectures deformed under simple shear, the effect of hinge elasticity on reinforcement is the most prominent under small strains when the bending of fibers is negligible (compare Figure 8c,e). At the shear strain of 0.02, the contribution of the rectandular layer to the shear force is close to zero at *k*_h_ = 0 (blue line in Figure 8e) and increases to 0.6 at *k*_h_ = *k*_b_ (blue line in Figure 8c).

For the hourglass and honeycomb scaffolds, the force contributions at a shear strain of 0.02 are comparable and are approximately equal to 0.35 for *k*_h_ = 0 (green and magenta lines in Figure 8e); these contributions to the force increase to 0.55 (green line in Figure 8c) and 0.9 (magenta line in Figure 8c) in the case of elastic hinges *k*_h_ = *k*_b_.

An increase in the shear strain results in increases in the force contribution, with the slope being dependent on the layer arrangement and the value of *k*_h_. At the maximal shear strain of 0.4 the force contributions of the three fiber arrangements at *k*_h_ = *k*_b_ are very close to those at *k*_h_ = 0 (Figure 8c,d), indicating that the prevailing forces are due to bending and stretching or compressing of the fibers, rather than the elasticity at the hinges. Note that the hourglass scaffold goes out-of-plane at higher shear strains in both the cases of freely jointed and elastic hinges (green lines and arrows in Figure 8c,e).

In contrast to the shear deformation, the elasticity of hinges contributes to the force ratios *F*_L_/*F*_tot_, under both small and finite tensile deformations (see Figures 8d,f). We note that under extension, the elastic hinges induce bending deformations of the interconnecting fibers (and hence, the corresponding force contributions) to minimize deviations of the hinge angles from their equilibrium values. The latter behavior is clearly seen from comparison of Figures S2b,d in the Supporting Information showing the top views of the rectangular fiber scaffolds under the tensile strain of 0.4 at *k*_h_ = *k*_b_ and 0, respectively. The freely joined fibers in Figure S2d remain mostly straight, whereas the fibers between elastic hinges are bent in Figure S2b. In the case of hougrass-shaped fiber layer, the transition to the out-of-plane configuration takes place only for elastic hinges and is absent at *k*_h_ = 0 (green lines in Figures 8d,f, green arrow in Figure 8d).

## Conclusions

Taking advantage of the deformation behavior displayed by certain regular arrangements of stiff fibers, we embedded the fibers into a polymer gel to determine how the geometry of the fiber layer (rectangular, hourglass and honeycomb) affects the resulting composite’s resistance to finite deformations and the stability of this sample’s shape. We developed a model that explicitly included features of swollen polymeric networks, accounting for the neo-Hookean elastic properties of the hydrogel and Flory–Huggins polymer–solvent interactions. Within the polymer matrix, the fibers contributed to the elasticity of the composite through the stretching and bending of these stiff segments and the deformation of the interconnecting hinges. Our computer simulations revealed cooperative interactions between the embedded stiff fibers and the gel matrix that led to the reinforcement under both small and finite deformations (shear and tensile), and to shape changes under finite tensile deformations.

The computer simulations showed that depending on the type of fiber arrangement, the gel-fiber composite could exhibit the auxetic behavior (negative Poisson’s ratio) in one or two directions. Specifically, the gel composite acquired auxeticity in two directions after embedding the inherently auxetic hourglass arrangement of fibers into the soft hydrogel matrix. The mismatch between the negative Poisson’s ratio of hourglass scaffold and the positive Poisson’s ratio of hydrogel matrix led to buckling of the scaffold and out-of-plane deformation of the gel-fiber composite under finite extensions. The hourglass scaffold also provided the least reinforcing effects among the three modeled arrangements because buckling of the scaffold reduced the stored elastic energy.

The best reinforcement of the composite was observed when the honeycomb scaffold was embed into the hydrogel. The Poisson’s ratio of the honeycomb scaffold is positive, displaying a value that is greater than that of the gel. Hence, the fiber layer and gel matrix stay in the respective stretched and compresses states under tension thus enhancing storage of elastic energy. The computer simulations showed that this gel-fiber composite exhibited specific shape changes under finite extension, with the shape changes being evident as the composite displays negative out-of-plane and positive in-plane Poisson’s ratios.

Under large deformation (at fixed temperature), the optimal mechanical properties were observed when the rectangular fiber arrangement was embedded into the gel. Among the three composite architectures considered here, the difference between the Poisson’s ratio for the rectangular scaffold and the gel exhibited the smallest value. The latter indicates similar shape changes of the two constituents under tension, and as a result provided the most stable composite shapes.

Notably, polymers are vital components in the production of such diverse systems as aeronautic components to biomimetics platforms and thin film displays. Our findings reveal the effective arrangements of embedded fibers to enhance the resilience and mechanical properties of the polymer matrices and thus yield superior composites for these technological applications.

## Supplementary Material



## Notes

Schematics of deformations (Figure S1); top and side views of finite deformations of gel sample having the fibers of rectangular arrangement embedded in the middle plane for elastic and freely joined hinge connections (Figure S2); comparison of finite deformations of gels having fiber layers embedded on the top surface and in the middle of gel (Figure S3); top and side views of finite tensile deformation of gel samples having the fiber layers attached to the top surface (Figure S4) (PDF)
