# Supplementary material for: Computer Simulations of Soft Responsive Gels with Embedded Regular Arrangements of Stiff Fibers
Source: Langmuir. 2026 Jan 13;42(3):2483–92. doi: 10.1021/acs.langmuir.5c04676 (PMC12856911; doi:10.1021/acs.langmuir.5c04676)
Supplement: Supplementary file 1 [file la5c04676_si_001.pdf]

Supplementary Information

Computer simulations of soft responsive gels with embedded regular arrangements of stiff fibers

Victor V. Yashin, Santidan Biswas, and Anna C. Balazs*
Chemical Engineering Department, University of Pittsburgh, Pittsburgh, PA 15261, United States

* balazs@pitt.edu

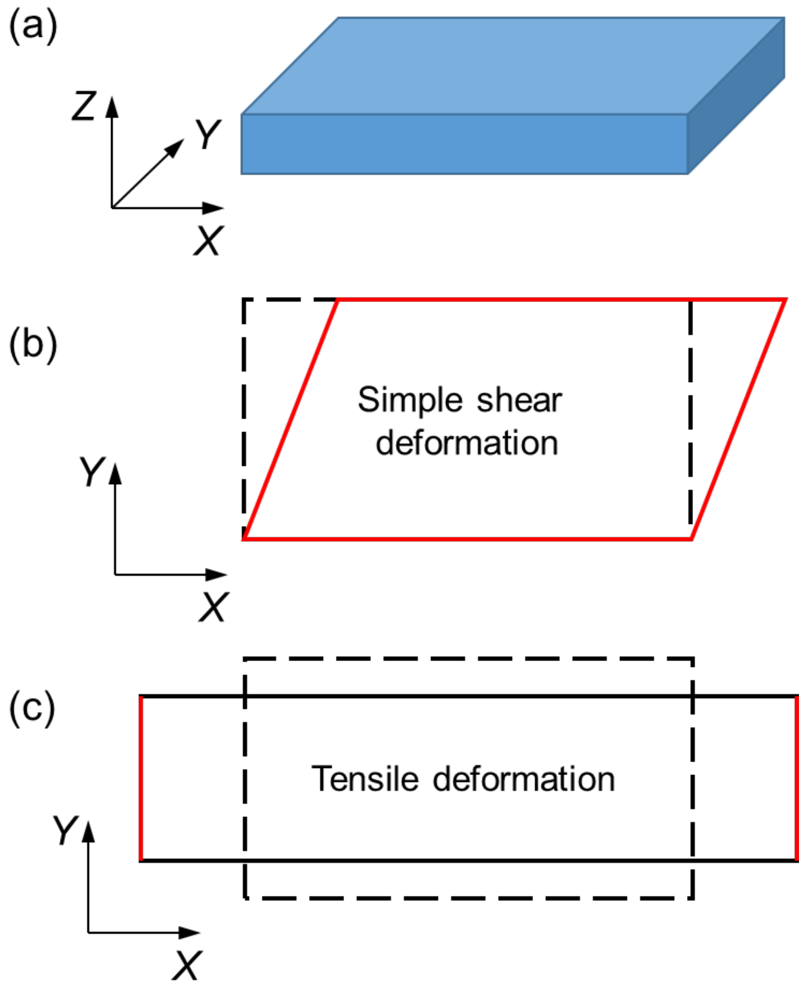

Figure S1.
Schematics of deformations. (a) Un-deformed sample. (b) Tensile deformation. (c) Simple shear deformation. Red-colored lined in (b) and (c) mark the side faces, which encompass fixed nodal points. For the simple shear deformation in (b), the nodes are fixed on all six surfaces including the top and bottom faces in the Z direction. In the case of tensile deformation in (c), only the X nodal coordinates are fixed on the left and right faces in the X direction, and the latter nodes can move in the YZ plane during relaxation.

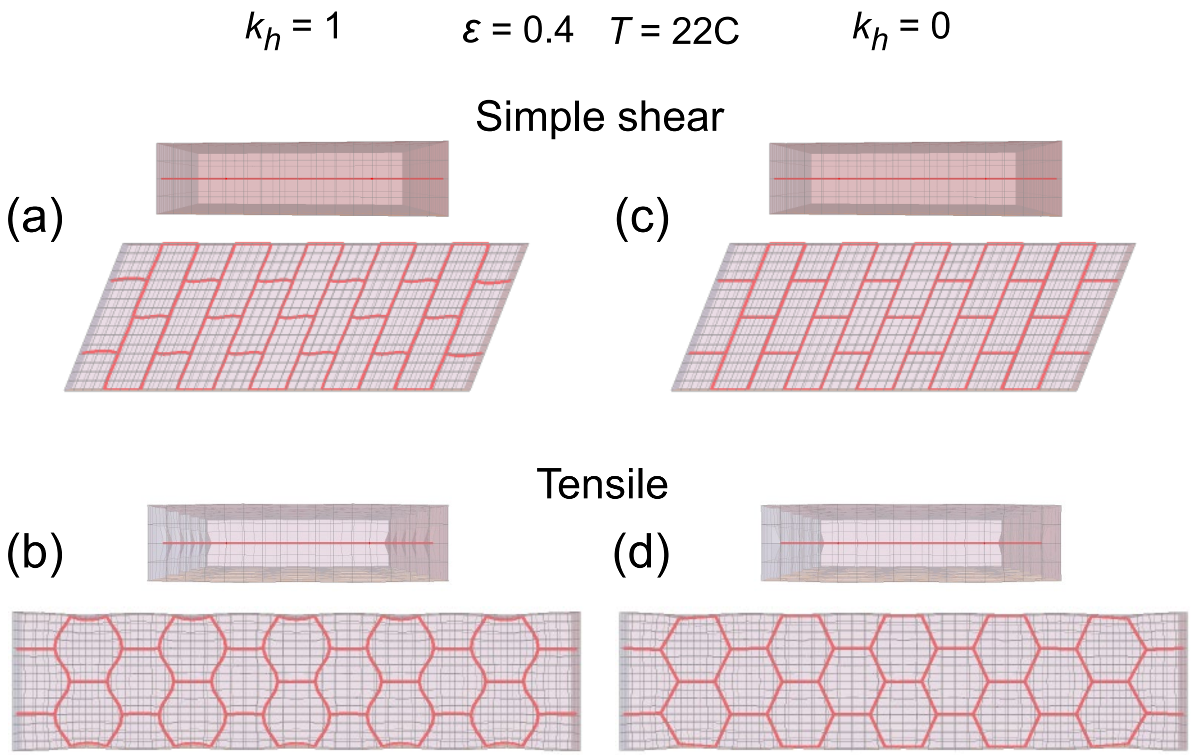

Figure S2.
Top and side views of finite (a), (c) simple shear and (b), (d) tensile deformations of gel sample having the fiber of rectangular arrangement embedded in the middle plane. Panel (a) and (b) correspond to the hinge elasticity of $k_h = k_b$, and (c) and (d) show the case of freely-jointed hinges at $k_h = 0$. Strain $\varepsilon = \varepsilon_{xx} = 0.4$. Temperature $T = 22°\mathrm{C}$. Note that there is no the out-of-plane deformation of the fiber layer in (a)-(d), and that the sample keeps the brick-like shape under the extension in (b) and (d). Note also bending of the fibers between the elastic hinges at $k_h = k_b$ in (a) and (b). Figures (a) and (b) are the same as Figs 6c and 6d.

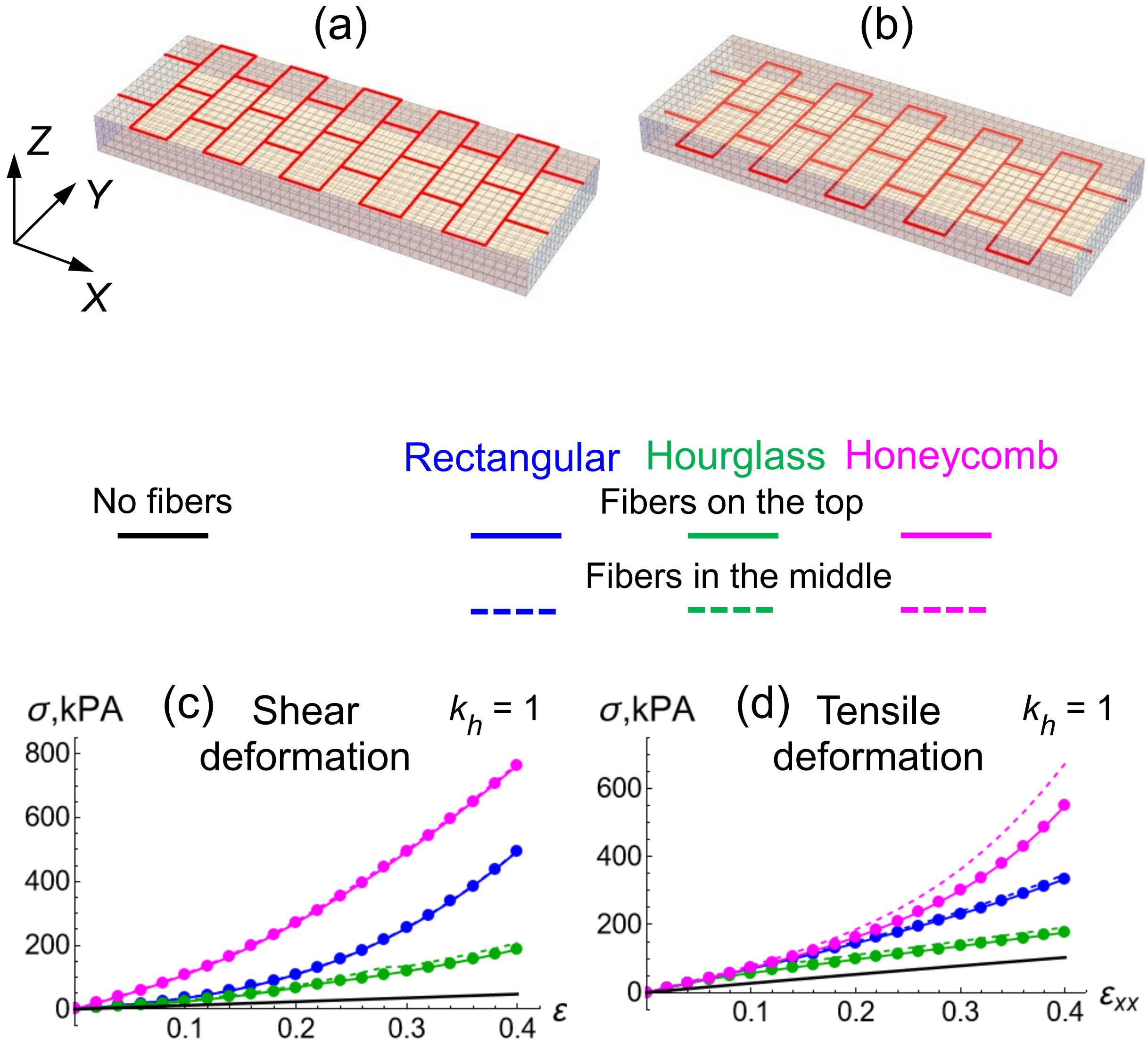

Figure S3.
Finite deformations of gels having fiber layers embedded on (a) the top surface and (b) in the middle of gel at the temperature of $T = 22°\mathrm{C}$ and the maximal strains of 0.4. The stress-strain curves for the finite (c) shear and (d) tensile deformations are shown for the fiber layers of rectangular (blue), hourglass (green) and honeycomb (red) arrangements under the elasticity of hinges of $k_h = k_b$, and for pure gel with no fibers (black). In (c) and (d), the symbols and solid lines denote the fiber layer on the top surface shown in (a), and the dashed lines correspond to the fiber layer in the middle of gel shown in (b). Note that in (c) and (d), the data for the fiber layer in the middle are the same as in Figs. 6a and 6b, respectively. In the case of fiber layers attached to the top surface in (a), the nodal coordinates are fixed only on the four side faces (Fig S1b) and the nodes are free to move in the all three dimensions on the top and bottom faces of the sample during relaxation.

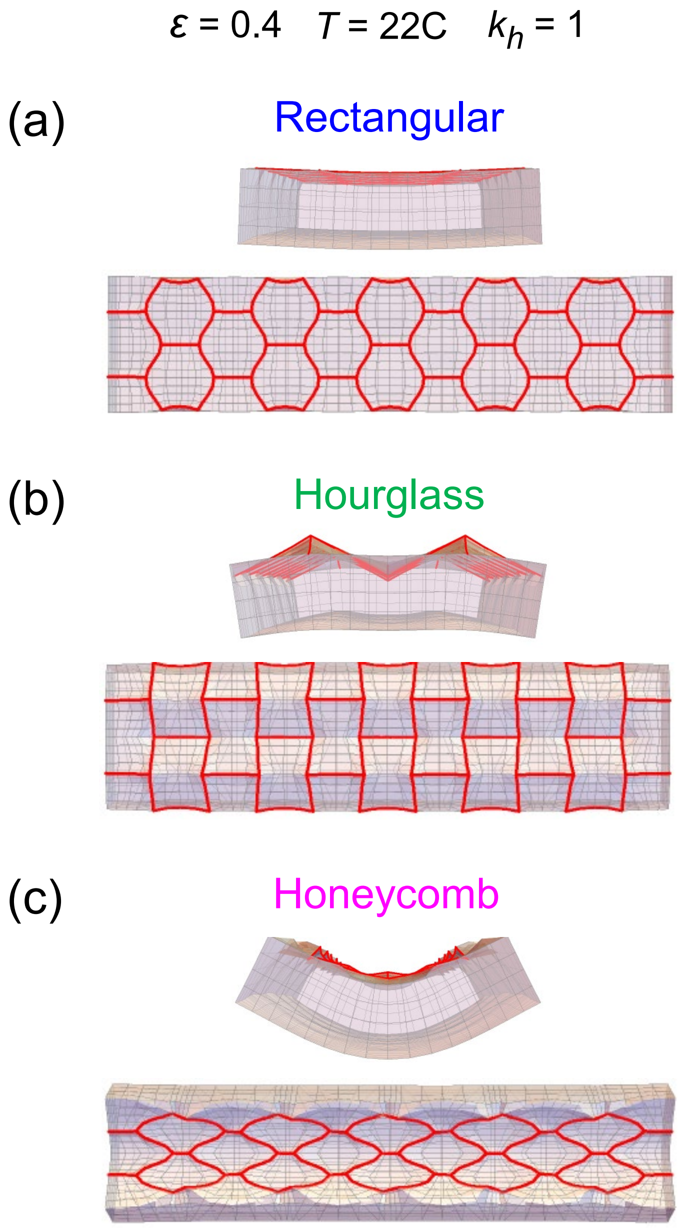

Figure S4.
Top and side views of finite tensile deformation of gel samples having the fiber layer of (a) rectangular, (b) hourglass and (c) honeycomb arrangements attached to the top surface under the hinge elasticity of $k_h = k_b$. Strain $\varepsilon = \varepsilon_{xx} = 0.4$. Temperature $T = 22°C$. Note that the sample with attached rectangular fiber layer in (a) retains almost brick-like geometry under extension, the hourglass layer in (b) is deformed out-of-plane and causes the gel matrix to bend upwards, whereas the honeycomb layer in (c) cases the gel to bend downwards under extension.
